# Single cell genomics yields a wide diversity of small planktonic protists across major ocean ecosystems

**DOI:** 10.1038/s41598-019-42487-1

**Published:** 2019-04-15

**Authors:** M. E. Sieracki, N. J. Poulton, O. Jaillon, P. Wincker, C. de Vargas, L. Rubinat-Ripoll, R. Stepanauskas, R. Logares, R. Massana

**Affiliations:** 1 National Science Foundation, 2415 Eisenhower Ave., Alexandria, VA 22314 USA; 2 Bigelow Laboratory for Ocean Sciences, 60 Bigelow Drive, East Boothbay, ME 04544 USA; 3 Génomique Métabolique, Genoscope, Institut de biologie François Jacob, Commissariat à l’Energie Atomique (CEA), CNRS, Université Evry, Université Paris-Saclay, Evry, France; 4 Sorbonne Universités, UPMC Université Paris 06, CNRS, UMR7144, Station Biologique de Roscoff, 29680 Roscoff, France; 5 Department of Marine Biology and Oceanography, Institute of Marine Sciences (ICM)-CSIC, Pg. Maritim de la Barceloneta, 37-49, Barcelona, E-08003 Catalonia Spain

## Abstract

Marine planktonic protists are critical components of ocean ecosystems and are highly diverse. Molecular sequencing methods are being used to describe this diversity and reveal new associations and metabolisms that are important to how these ecosystems function. We describe here the use of the single cell genomics approach to sample and interrogate the diversity of the smaller (pico- and nano-sized) protists from a range of oceanic samples. We created over 900 single amplified genomes (SAGs) from 8 *Tara* Ocean samples across the Indian Ocean and the Mediterranean Sea. We show that flow cytometric sorting of single cells effectively distinguishes plastidic and aplastidic cell types that agree with our understanding of protist phylogeny. Yields of genomic DNA with PCR-identifiable 18S rRNA gene sequence from single cells was low (15% of aplastidic cell sorts, and 7% of plastidic sorts) and tests with alternate primers and comparisons to metabarcoding did not reveal phylogenetic bias in the major protist groups. There was little evidence of significant bias against or in favor of any phylogenetic group expected or known to be present. The four open ocean stations in the Indian Ocean had similar communities, despite ranging from 14°N to 20°S latitude, and they differed from the Mediterranean station. Single cell genomics of protists suggests that the taxonomic diversity of the dominant taxa found in only several hundreds of microliters of surface seawater is similar to that found in molecular surveys where liters of sample are filtered.

## Introduction

Planktonic protists in the surface ocean are ubiquitous, abundant and highly diverse. They range in size from the smallest known eukaryote, *Ostreococcus* spp. (0.8 µm)^1^, to large ciliates, radiolarians, and protist colonies 100’s of micrometers across and visible to the naked eye. They function as primary producers, grazers and parasites, and influence the packaging and recycling of carbon and nutrients in marine ecosystems. Protists associate with prokaryotes, sometimes endosymbiotically, to conduct fundamental biogeochemical transformations such as nitrogen fixation^2^. Despite this ecological importance in the structure and function of marine ecosystems the smaller planktonic eukaryotes are not as well characterized as the larger microplankton due to their small size, lack of distinctive morphological features, and the lack of cultures of many dominant forms, especially of the aplastidic bacterivorous protists^3^.

As with prokaryotes, genetic methods have revealed remarkably diverse ocean planktonic protist communities^4^. These methods include direct cloning of environmental DNA, fingerprinting methods, tag sequencing, and metagenomics of filtered or sorted fractions of the community. These methods have various advantages and disadvantages depending upon the science question being addressed^5^. For assessing the diversity of the dominant forms present in seawater, clone libraries and tag sequencing have been the favored approaches. These methods have the disadvantage of being biased in favor of particular, often larger, cell types, which can have 10’s to 100’s of copies of target genes per cell (in particular the 18S rDNA^6^) and thus are artificially overrepresented in these surveys^7^. This seems to be especially true of the alveolates, including marine alveolate groups I and II, dinoflagellates, and ciliates. Fine plankton nets and filter fractionation is often used to characterize plankton communities, but these can break up fragile animals, colonies, and individual cells, sending their DNA into small size fractions^8^.

It has been known for some time that many marine protists are mixotrophic and are not easily assigned to photo- or heterotrophic categories^9,10^. More recent results confirm that many of the small planktonic chlorophyll-containing cells are mixotrophs, ingesting bacteria^11,12^. Flow cytometry easily distinguishes cells containing chlorophyll from those that do not by the presence of chlorophyll autofluorescence. We, therefore, use the terms “plastidic” and “aplastidic” here to distinguish the presence or absence of chloroplasts, without assigning a trophic category to them. In this nomenclature plastidic cells are most likely phototrophic or mixotrophic, although some could also be heterotrophs with a phototroph in their food vacuole. Aplastidic cells are most likely to be heterotrophic, but could be phototrophs or mixotrophs with reduced chloroplasts or faint chlorophyll fluorescence.

The single cell approach has proven its power in the discovery of new potential metabolisms in uncultured prokaryotes^13^, and has the advantage of yielding large amounts of genomic DNA from individual microorganisms for further sequencing and investigation. Early results from one coastal Maine sample revealed significantly higher protist diversity in whole water samples using the single cell approach compared to clone libraries^7^. The bias due to gene copy number in the clone libraries was the likely cause of the underestimation. Sequencing of three “picobiliphyte” (now Picozoa) SAGs from that sample showed how this approach can reveal microbial interactions between eukaryotes, prokaryotes and viruses^14^.

Here we report on a larger set of protist SAGs collected across a wider range of ocean samples for single cell genomics on the *Tara* Oceans expedition^15^. We analyzed the 18S rDNA sequences of over 900 SAGs from the Adriatic Sea, Arabian Sea and the Indian Ocean across a wide latitudinal gradient. A subset of these SAGs has recently been more fully sequenced and assembled^16–19^. These studies demonstrate that, although individual SAGs may represent only a portion of the cellular genome, the co-assembly of multiple SAGs can improve genome recovery significantly. For MAST-4 clade, the co-assembly of 14 SAGs yielded ~74% of genome recovery^16^, and for *Monosiga brevicollis* four co-assembled SAGs yielded 46% of the genome^17^. Single cell genomes were used as reference genomes to match with metagenomic data and reveal biogeographic patterns of *Bathycoccus*^18^, and unexpected functional diversity of the dominant MAST-4 heterotroph clade^19^. The work reported here shows that our sampling and cell handling approach appears to accurately sample the diversity of the dominant oceanic eukaryotes in the pico- to nanoplankton size range (<5 µm).

## Methods

### Cryopreservation and flow cytometric detection

Tests were done to confirm and optimize the cryopreservation of marine protists for single cell genomics using marine samples from 1 m depth at the dock in Booth Bay, Maine, a coastal Atlantic site. The cryoprotectant glycine betaine^20^ had previously proven to preserve prokaryotic cells, allow identification of nucleic acid stained cells by flow cytometry, and not interfere with single cell amplification, PCR screening, and sequencing reactions^21^. Live (aplastidic) protists had been sorted and successfully amplified and PCR screened using light scatter properties and Lysotracker staining^7,22^. Lysotracker, however, only stains live cells with active vacuoles and does not stain cryopreserved cells. As an alternative, we adopted the method of Zubkov, *et al*.^23^ based on SYBR Green I staining for detecting aplastidic cells. Plastidic cells are easily distinguished by red autofluorescence of chlorophyll emitted by chloroplasts using flow cytometry. An experiment was conducted to compare the cell numbers of aplastidic cells obtained by flow cytometry using both fresh and cryopreserved samples with the cryoprotectants glycine betaine (GBe, 7% v/v, Sigma) and glycerol-TE (Gly-TE, 5% glycerol + 1x TE buffer, Sigma). Counts of the cryopreserved samples were determined after staining with SYBR Green I (1:5,000 dilution; ThermoFisher Scientific, USA), while counts of fresh samples were determined after samples stained with both SYBR Green I (SYBR, 1:5,000 dilution; ThermoFisher Scientific, USA) and Lysotracker (LT, 75 nmol; ThermoFisher, Scientific, USA).

### Ocean sampling

Whole water samples were taken from surface ocean water, or from the deep chlorophyll maximum (DCM), by a submerged impeller pump. Sample sites included the Adriatic Sea, Arabian Sea and the Equatorial Indian Ocean. Subsamples were dispensed into replicate 4 mL cryovials containing GBe as a cryoprotectant (7% w/v, final conc.). The cryovials were flash frozen and stored in liquid nitrogen (LN) until SV Tara reached a shipping port.

Hydrographic data, including salinity and temperature, was determined at each station using a CTD with a bottle rosette onboard SV Tara. Bottle samples were analyzed for chlorophyll by HPLC, and for counts of the small cells using standard flow cytometry methods^24^.

### Single cells

Samples were express-shipped on dry ice to Bigelow Laboratory for Ocean Sciences where they were stored in LN until sorting. SAG generation and identification were performed at the Single Cell Genomics Center at Bigelow (scgc.bigelow.org). On the sorting day tubes were thawed at room temperature and a subsample was stained with SYBR Green I. Sorting was conducted on a Beckman-Coulter MoFlo sorter outfitted with a Cyclone™ robotic arm for sorting into plates. Single plastidic cells were sorted using the natural chlorophyll autofluorescence within an unstained subsample and single aplastidic cells were sorted using a SYBR Green I (1:5000 dilution) stained subsample^23^. All single cells were sorted into 384 well plates containing 0.6 µL TE buffer per well. Multiple plates were prepared for unstained plastidic and stained aplastidic cells from each sample. After sorting, all plates were stored frozen at −80 °C.

### Lysis and MDA

Attempts to improve the amplification yield of single cells were made by increasing the number of freeze-thaw cycles and incubating with KOH at 20 °C. Incubating with KOH at higher temperatures resulted in lower yields, probably due to DNA degradation. We settled on 5 cycles of freeze-thaw as optimal.

Genomic DNA from single cells was amplified using the phi-29 polymerase (real-time multiple displacement amplification, rtMDA) method in 384-well format^13^. Amplification reactions were run overnight (ca. 18 h) and monitored in real time based on DNA fluorescence. Critical point (Cp) values for each well were determined as the reaction time when well fluorescence reached half the maximum value. Based on these Cp values we selected those SAGs having Cp values below 14 h for further analysis.

### PCR screening

The genomic DNA produced by MDA served as template for screening using universal 18S rRNA gene eukaryotic PCR primers. All wells were screened regardless of their MDA Cp values. Primers used were Euk528 (forward)^6^ and Euk B (reverse)^25^ which amplify two thirds of the gene (ca. 1200 bp)^26^. PCR amplicons were sequenced using Sanger technology using the same two primers. Sequences were curated manually and compared to sequences in GenBank using BLAST to determine similarity to known sequences. Closest matches and closest cultured matches were recorded. Sequences were aligned using MAFFT and compared to each other, and to reference sequences for some groups, using maximum likelihood trees (RAxML) to achieve a final phylogenetic assignment. To assess primer bias in sampling protistan diversity we additionally screened one plate each of plastidic and aplastidic protists from one sample, Stn 41 surface, with two additional primer sets targeting the variable V4 and V9 regions of the rRNA gene (see PCR protocols and primer sequences in refs.^27,28^, for V4 and V9 regions, respectively).

### Comparison of SAG sequences with metabarcoding data

We compared the relative community composition at three *Tara* Oceans stations using available V9 metabarcodes at the group level with the SAG samples. Detailed information on sampling and metabarcoding (iTAG) sequencing can be found in Pesant *et al*.^29^ and de Vargas *et al*.^28^, respectively. We separated the iTAG sequences into plastidic and aplastidic types by assigning them to class-level groups, removing groups not targeted in the SAGs such as ciliates, diatoms, dinoflagellates, MALVs, radiolarians and unassigned. Then the proportions were calculated on the remaining 33 groups for comparison with SAGs.

We also used the V9 metabarcodes obtained from Tara Oceans samples to explore the occurrence and abundance of SAGs in the global ocean. For this analysis, we only considered samples obtained from the photic zone (surface and DCM) and the smaller size fractions, piconano- (0.8–5 µm) and nano- (5–20 µm) sized cells. We ended up with a dataset containing barcodes from 337 samples deriving from 105 stations. The resulting metabarcode table had 435,240,095 V9 sequence reads grouped into 4,298,066 valid barcodes. The barcodes were clustered into OTUs using SWARM 2.1.129 with default options (local clustering threshold d = 1), generating a total of 271,787 OTUs^28^. We mapped the V9 sequences of 868 SAGs on these OTUs using BLAST 2.6.0 and selected the 671 hits that were retrieved with similarity >97% and coverage >80%.

For comparison, we also mapped the Marine Microbial Eukaryote Transcriptome Sequencing Project (MMETSP)^30^ V9 sequences onto these oceanic OTUs. Among the 385 MMETSP transcriptomes for which we could recover sequences of the V9 region, 212 were mapped on some OTU at similarity >97% and coverage >80%.

## Results

### Sample locations and water properties

Sample locations, water properties, and number of recovered SAGs are shown in Table 1. All stations are open water except for station 46, which was in the middle of a tropical lagoon (Supplementary Fig. S1).

**Table 1:** Samples station locations, dates, water characteristics, pico- and nanoplankton cell abundances, and numbers of plastidic and aplastidic SAGs obtained.

								Chl			Plastidic euks		No. of SAGs	
Stn	Date mm/dd/yy	Site^a^	Lat (deg. N)	Lon (deg. E)	Depth (m)	Temp. (°C)	Salinity (psu)	µg L^−1^ *(s.e.)*	*Syn* # mL^−1^	*Pro* # mL^−1^	Small # mL^−1^	Large # mL^−1^	Plast.	Aplast.
23	11/18/09	Ad	42.18986	17.71670	55	17.32	38.201	0.139 *(0.04)*	10,448	19,390	392	699	24	118
39	03/18/10	Ar	18.57138	66.53050	S	26.82	36.285	0.099 *(0.02)*	146,758	125,121	3,275	2,165	38	52
41S	03/30/10	IO	14.59540	69.98100	S	29.09	36.025	0.020 *(0.02)*	13,703	119,176	3,507	1,393	57	88
41D	“	IO	“	“	59	27.21	36.499	0.373 *(0.11)*	3,809	245,757	888	nd	64	141
46	04/15/10	M	−0.66245	73.16097	S	30.13	35.111	0.122 *(0.01)*	178,299	157,827	10,607	596	72	78
47	04/16/10	IO	−2.04653	72.15680	S	30.20	34.912	0.007 *(0.01)*	210,569	1,398	677	677	26	26
48	04/19/10	IO	−9.40295	66.36804	S	29.83	34.175	nd	497	119,104	498	nd	37	21
51	05/11/10	IO	−21.50212	54.35328	S	27.26	34.901	0.040 *(nd)*	1,336	221,147	653	nd	35	26

The small and large plastidic eukaryotes were identified and counted by flow cytometry triggered on chlorophyll autofluorescence, so aplastidic protists are not counted here.

^a^Ad = Adriatic Sea, Ar = Arabian Sea, IO-Indian Ocean, M = Addu Atoll, Maldives, s.e. = standard error, S = surface (<3 m), sample depths greater than 50 m were targeted at the subsurface chlorophyll maximum, nd = not detected.

### Cryopreservation

**Figure 1:**
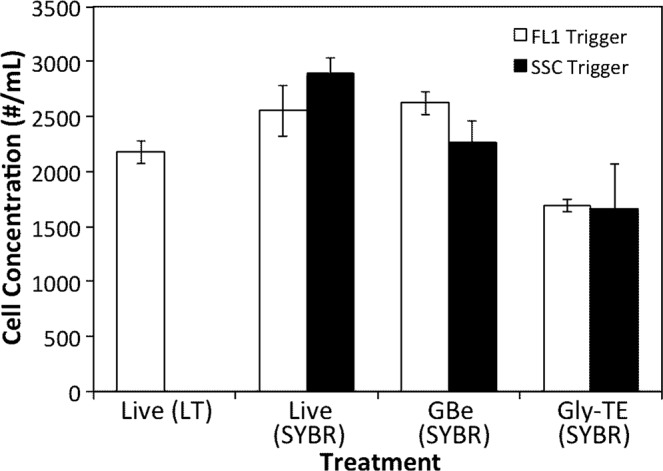
Comparison of flow cytometric cell counts of aplastidic protists counted live and with two cryoprotectants. Triplicate samples of coastal Maine water were run live and stained with either Lysotracker (LT) or SYBR green. Replicate samples were stored with the cryoprotectants glycine betaine (GBe) or glycerol – TE (Gly-TE) at −80 °C and then stained with SYBR green before enumeration. Each replicate was run with the flow cytometer triggered by green fluorescence (FL1) or by side scatter (SSC). Errors bars show standard deviations of triplicate samples.

Preliminary tests using the cryoprotectant glycine betaine (Gbe) indicated that this method worked well for sorting and single cell genomics of protists. Chlorophyll fluorescence was preserved in the plastidic cells for discrimination by flow cytometry (Supplementary Fig. S2). Aplastidic cells preserved this way could be stained using SYBR Green I and a sort region was created similar to that in Zubkov *et al*.^23^ (Supplementary Fig. S2). We then compared cryopreservation with the live staining methods for cell counts of aplastidic protists, and found that the GBe method showed the lowest cell loss compared to live samples, or the other cryoprotectant glycerol-TE (glyTE) (Fig. 1). In fact, live cell counts determined by SYBR Green I staining were higher than by Lysotracker staining, and the counts in the GBe cryotreatment were not significantly different from those using SYBR Green I stained cells.

### MDA and PCR performance

Initial rtMDA results for the protists yielded fewer positive wells (<20%) than what we usually observe for oceanic prokaryotes (average 27%)^31^. We tried different lysis protocols including multiple freeze-thaw cycles and increasing the temperature of the 10 minute KOH incubation, but these simple modifications did not improve yield significantly (data not shown).

Generally we found that plastidic cells had a lower yield of good quality 18S rRNA gene sequences than aplastidic cells (Supplementary Table S1). Twenty-nine plates were processed and analyzed, comprising 9,135 one cell wells (non-controls). We recovered good quality 18S rRNA gene sequence from 7.2% of the plastidic cell wells, and from 14.7% wells of aplastidic cells. These were statistically different at a confidence level of 95% (Student’s t-test, *p* = *0.046*). There are 3,408 one-cell wells (37%) with good MDA, but no 18S rRNA gene identity. These wells are likely to contain amplified eukaryote genomic DNA, but where 18S rRNA genes could not be recovered due to uneven MDA, PCR primer mismatches, long inserts in 18S rRNA genes, or other interferences^32^. Conversely, there were some wells (124, 1.4%) with good 18S rRNA gene identity but with poor MDA (Cp > 14 h). These could contain a limited amount of genomic DNA. The list of SAGs with good 18S rRNA gene identity is given in Supplementary Table S2. Sequences have been submitted to the European Nucleotide Archive (ENA, accession numbers PRJEB31452).

### V4–V9 primer screens

The numbers of SAGs identified by each of the three primer sets used showed that the addition of the V9 primer screen identified 11 additional SAGs from each plate tested, one plastidic and one aplastidic sort (Supplementary Fig. S2). The addition of V4 only identified six more SAGs from the plate of plastidic cells, and three more from the aplastidic plate. Conversely, the V4 primers missed 23 SAGs (13 plastidic, 10 aplastidic) that were identified by the Euk528/B primer set, and the V9 primers missed 16 SAGs (8 from each plate). There were no major new groups that were picked up by the new primers in these plates.

### Distribution of sorted cells across phylogenetic groups

**Figure 2:**
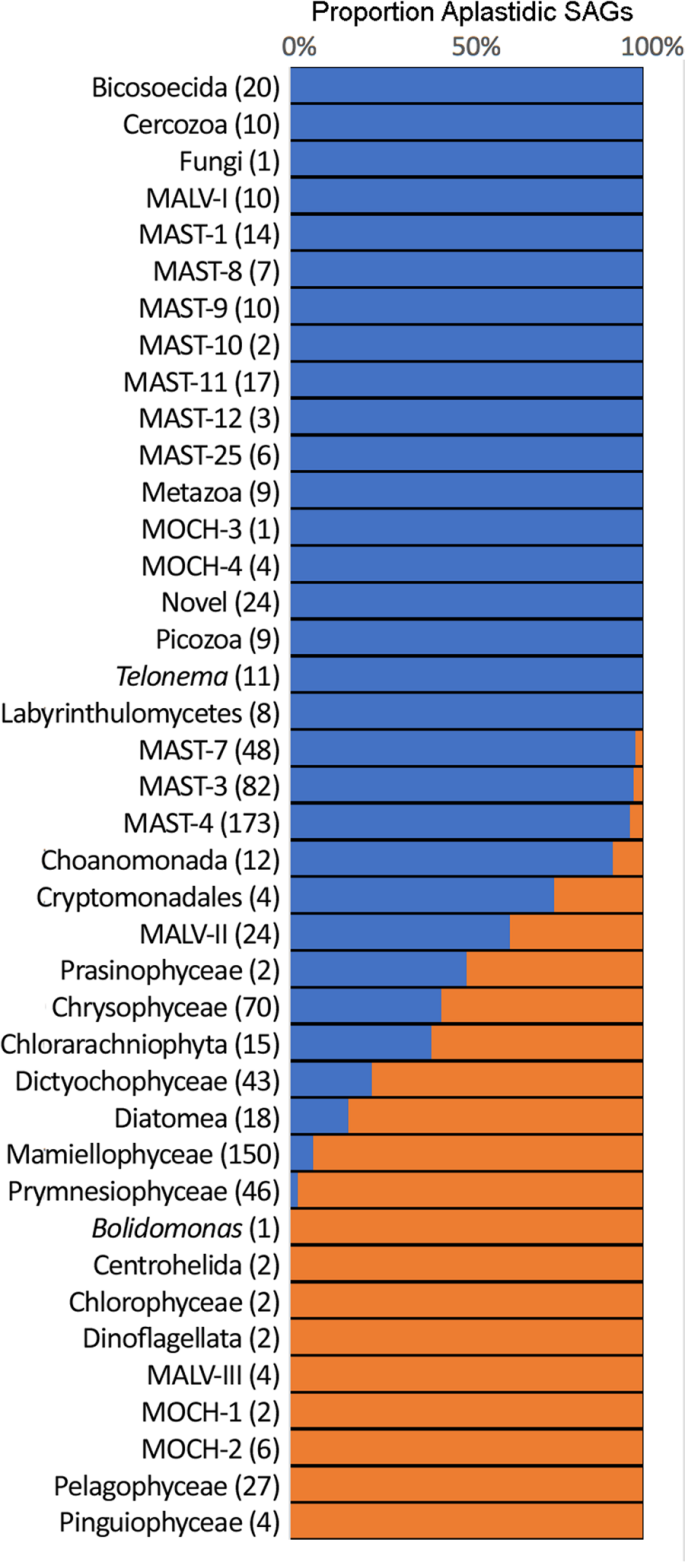
Distribution of plastidic and aplastidic SAGs within the major taxonomic groups found. The bars show the proportion of SAGs in each group that were sorted as aplastidic (blue bars) and plastidic (orange bars) cells. The groups are ranked by proportion and the number of identified SAGs for each is given in parentheses.

The distributions of plastidic and aplastidic cells, as determined by flow cytometry (i.e. presence or absence of chlorophyll fluorescence), is shown in Fig. 2 for the defined taxonomic groups. Twenty-two of these groups were represented essentially by aplastidic cells and twelve of them by plastidic cells. Interestingly, some of the groups represented by cells from both sorts (i.e. Chrysophyceae, Chlorarachniophyta, Dictyochophyceae) are also well known for containing both plastidic and colorless species. More intriguing was the presence of MALV-II among the two sorts. Generally, the distribution of chloroplasts across these groups is as expected based on what we know about their phylogeny and evolution^33^.

### Protist communities

**Figure 3:**
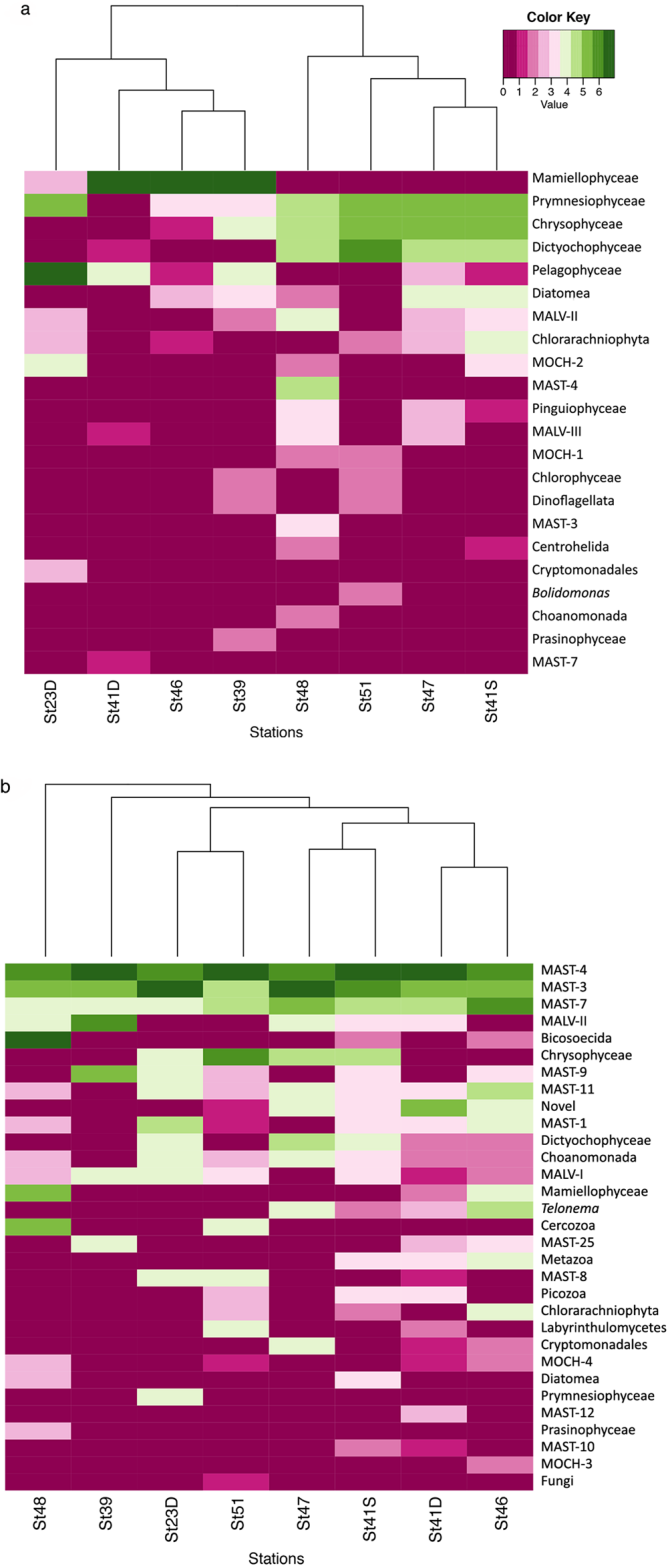
Heat maps showing the SAG composition of the (**a**) plastidic community and (**b**) aplastidic community at each station. The values on the color scale are the number of SAGs of each type transformed by log_2_(x) + 1 (with zeros left as zeros)^40^.

The protist communities recovered by the SAG approach were quite diverse in most samples (Fig. 3). Richness, calculated at the level of the groups defined here, was highest for station 41 surface, and lowest at stations 23, 39, and 46, while diversity (Shannon H) was highest at station 51, and evenness was highest at station 48 (Supplementary Table 3).

The plastidic cells for three stations, 39, 41 deep chlorophyll maximum, and 46, were dominated by Mamiellophyceae (Fig. 3a), specifically mixed blooms of *Micromonas* and *Ostreococcus spp*. with identical 18S rRNA sequences. Station 23D, in the Adriatic, was distinctive in that Pelagophyceae dominated the plastidic cells. The remaining stations (48, 51, 47 and 41S) had more diverse and similar community compositions with a mix of Prymnesiophyceae, Chrysophyceae, and Dictyochophyceae dominating.

The aplastidic protist communities were more similar to each other than the plastidic communities across stations at the taxonomic levels chosen (Fig. 3b). Overall three marine stramenopiles types, MAST-4, 3 and 7, made up about 50% of the aplastidic cells. Other dominant types of the aplastidic community across the other stations were Chrysophyceae, Bicosoecida, MALV-I, MALV-II, Telonema, Dichtyochophyceae and Picozoa. There are 9 cells that are from metazoans, mostly ctenophores and salps. One metazoan SAG with a novel 18S rRNA gene was found and appears to be from an acorn worm. Metazoan SAGs could have come from single cells from damaged animal tissue, fecal material, or as free-swimming gametes.

**Figure 4:**
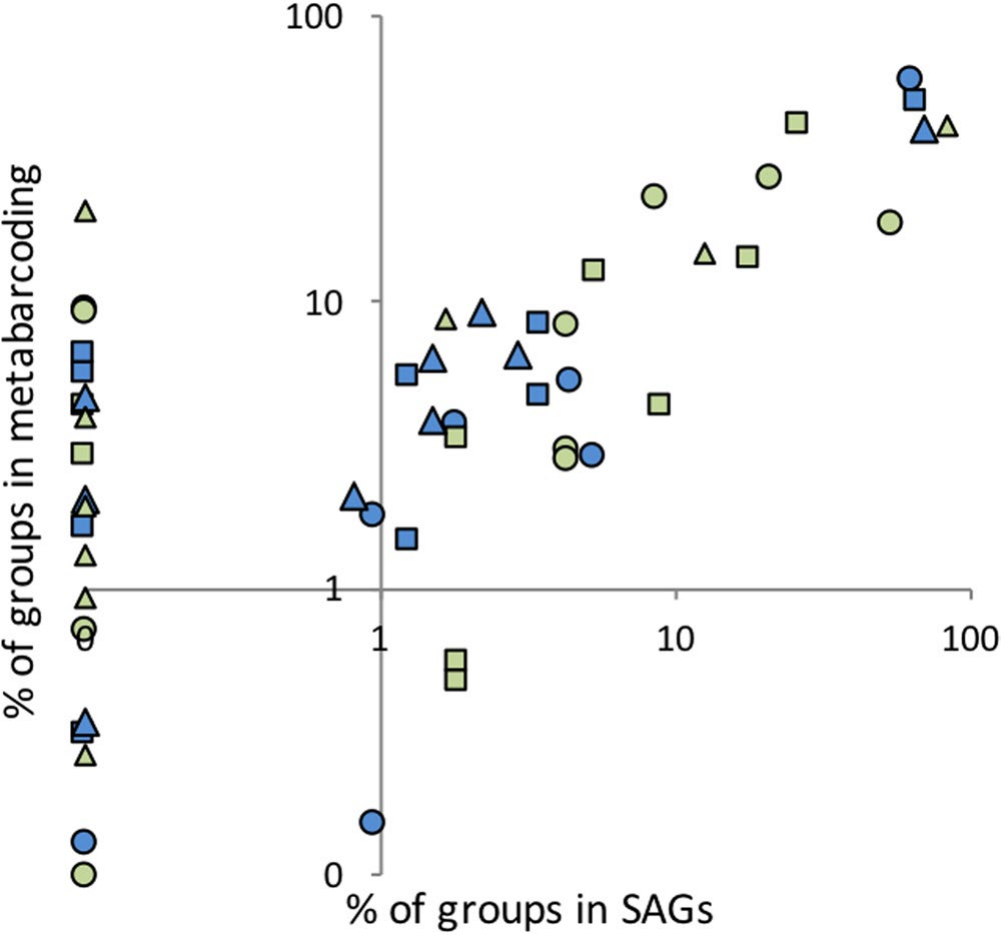
Comparison of the relative abundance of taxonomic groups found in the SAG collections and in metabarcoding dataset (iTAGs) at three stations for plastidic (green symbols) and aplastidic (blue) protists. Each station has a different plot symbol: circle (23-D), square (41-S), and triangle (41-D).

**Figure 5:**
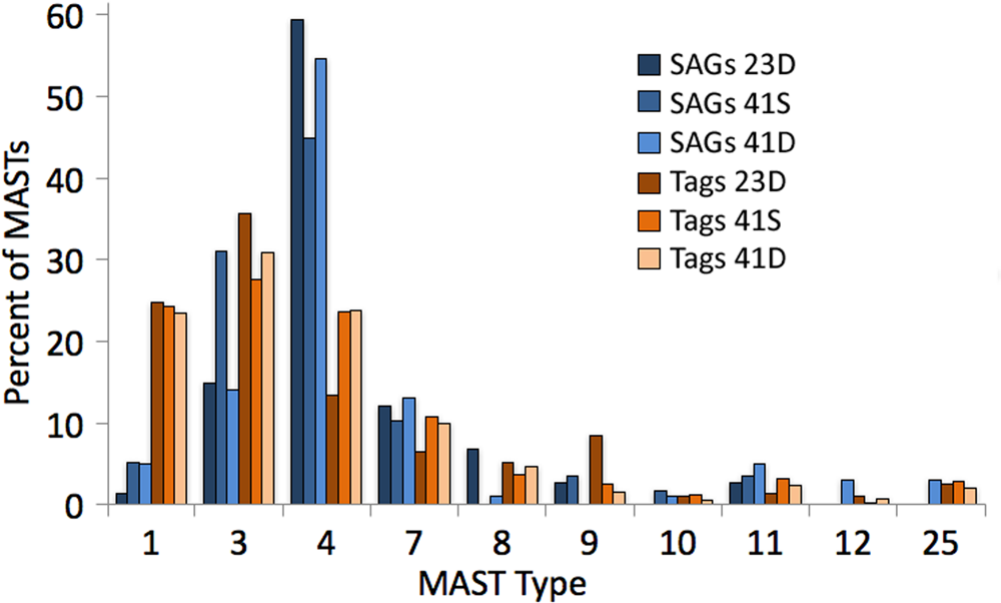
Comparison of the proportion of types found in SAGs (blue columns) and metabarcoding (iTAGs, orange columns) at three stations (23, 41S and 41D, represented by the different color shades) for the various MAST clades. Data calculated as percent of total MASTs.

The analysis of iTag sequences^28^ from the 3 samples where we could directly compare, revealed a general positive trend with the SAG groups with the exception of some groups found by the iTag method, but not in the SAGs (Fig. 4). The metabarcode (iTag) approach revealed groups that were not detected in the SAGs (points on the left of Fig. 4). This is likely due to differences in sampling between the two methods, most importantly the fewer cells identified per sample through the SAG approach. The SAGs were derived from small, whole seawater samples (ca. 300 microliters subsampled from several milliliters), whereas the metabarcoding samples were comprised of many liters of seawater size fractionated and collected on filters. For the SAG samples only the small, and most dominant protists were chosen for sorting. Analysis of the outliers - groups that were relatively underrepresented in the SAG data - was not particularly enlightening (data not shown). In the comparison of only marine stramenopile groups (Fig. 5) showed a similar distribution between the methods.

**Figure 6:**
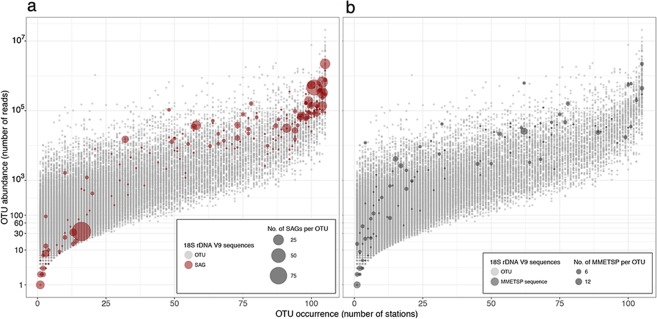
Mapping of SAG (**a**) and MMETSP (**b**) V9 sequences onto the Tara Oceans metabarcode V9 OTUs. The size of the colored bubbles represents the number of matching SAG or MMETPS sequences in each OTU. SAGs dataset represents a number cosmopolitan and abundant taxa while the MMETSP sequences have fewer matches and are spread across the range of dominance distribution.

The comparison of the SAG sequences against oceanic metabarcoding V9-swarms (Fig. 6a) shows that the SAGs represent the most abundant types (bubbles in the upper right) presumably corresponding to ecologically important groups. Some matches were also found to types rarer in the metabarcodes (Fig. 6b, lower left). In contrast to the SAGs, the sequences in the Marine Microbial Eukaryote Transcriptome Sequencing Project database (Fig. 6b) were more representative of rare types in the oceanic metabarcodes, with fewer matches in the upper right compared to the SAGs (Fig. 6a).

## Discussion

We have found that single cell methods developed to preserve samples and amplify genomic DNA for planktonic prokaryotes were generally transferrable to eukaryotes. The major difference is that Gly-TE, although a preferable cryoprotectant for prokaryotes, appears to be less effective for protists than GBe. We noticed that the yields of SAGs from protist plates, especially for the plastidic types, were lower than what we usually observed for planktonic prokaryotes. Our attempts to make simple modifications to our lysis methods failed to significantly increase yields of SAGs. The test of additional PCR primers to identify positive cases did yield some more identified SAGs, but did not reveal whole new cell types not seen with the Euk528/B primer set. We conclude from this test that the use of these additional primer screens slightly increased yield, but not diversity.

We observed that more of the cells sorted as aplastidic yielded successful MDA product than those sorted as plastidic. This could be due to lower lysis success with these groups, or interference with the MDA reaction by constituents such as polysaccharides, either within the cells or on the cell wall of plastidic cells. While the single cell approach avoids some biases, there may be others that affect our results. These include a possible lysis bias with some cell types being less likely to be opened and their genomes available for amplification than other types.

The sorting strategy using plastid autofluorescence was very effective, as the majority of the groups were sorted either in aplastidic or in plastidic sorts (not both, Fig. 1). Groups containing a mix of plastidic and aplastidic cells (Fig. 1) could be explained by several factors. Some groups (e.g. Choanomonada, MAST-3, and -4) are predominantly aplastidic bacterivores with only a few instances of plastidic types. These could be herbivores with a recently ingested plastidic cell^19^. For instance, it has been seen that MAST-4 is able to graze both on bacteria and plastidic picoeukaryotes^33^. In this case the fluorescence in their food vacuoles would cause it to be classified as plastidic. Other groups (e.g. Diatomea, Mamiellophyceae, and Prymnesiophyceae) are predominantly plastidic phototrophs where some cells might have very weak autofluorescence (little chlorophyll) and were not detected as plastidic. More interesting were the groups with similar numbers of aplastidic and plastidic cells (e.g. MALV-II, Chrysophyceae, Chlorarachniophyta and Dictyochophyceae) as most of these groups are known to contain plastidic and aplastidic species. They can also include species with weak fluorescence plastids or that change the pigment content depending upon circumstances. We may also have sorted infected autotrophs with degraded host nuclear DNA. The presence of the putative MALV-II parasite within this category is intriguing and deserves further analyses.

In this set of protist SAGs we found 9 Picozoa cells^34^ (formerly Picobiliphytes^35^), all in aplastidic sorts. This continues to confirm our observations of these organisms from Booth Bay, Maine^7^, where these types only appeared in sorts of cells without chlorophyll fluorescence. Yoon *et al*.^14^ found no genetic evidence of plastids in the partial genomes of three SAGs, and Seenivasan *et al*.^34^ obtained the first picozoan culture and found no evidence of plastids in serial thin sections.

There are a variety of factors that can bias our determination by the single cell approach of the community composition of marine microeukaryotes^7^. Koid *et al*.^5^ found that diatoms appear to be underrepresented in clone libraries, likely due to difficulties in lysing the cells and releasing the genomic DNA. Amacher *et al*.^36^ noted biases in clone libraries related to abundances of both target and co-occurring species. In our results we obtained 18 diatoms: 15 out of 353 plastidic SAGs, and an additional 3 from the 550 aplastidic SAGs. This might seem a small number, but we targeted a flow cytometric region that only contained small cells (about 2–5 µm in size) and diatoms are generally larger than this. Therefore, it is not clear if we missed diatoms due to inefficient lysis or because they were not included in the sorting gates. At any rate, the data shown here reveals that the community composition derived from the analysis of dozens of SAGs per sample is comparable with the more common metabarcoding molecular surveys.

The community composition of protists we observed in the Indian Ocean (Fig. 3) has some similarities and differences from previous observations based on clone libraries^37^. On a cruise from the southwest to the northeast Indian Ocean, adjacent to the Tara Oceans Expedition, reaching similar latitudes (25°–12°S) east of our transect, clone libraries of the cells passing a 3 µm filter revealed a somewhat different pattern of community structure than what we observed at our comparable stations (41, 47, 48 and 51)^37^. They found higher proportions of dinoflagellates, marine alveolates (esp. MALV-I), and radiolarians than we observed. They also found lower proportions of MAST, Chrysophytes, Dichtyochophytes, and Prymnesiophytes. Prasinophytes were common in both studies. The major differences between these studies seem to relate to the biases we have seen in clone libraries due to gene copy number. The types overrepresented in the Not *et al*.^37^ study relative to this study are types known to contain many copies of the targeted rDNA operon.

Assembling whole genomes from SAGs is generally difficult, and gets particularly challenging for eukaryotic genomes, which can be complicated by heterozygosity, and putatively massive repeated regions. New information can be obtained from partially assembled genomes, however, especially from uncultivated cell types^14,16–19,26^. Due to the nature of MDA, some sections of the genomic DNA are over amplified while other sections may not be amplified at all^32^, preventing sequencing and assembly of complete genomes from single cells. This appears to involve stochastic processes when amplifying a single DNA molecule as a starting template, as well as a bias against sequences with high G + C content^31^. Recent work has shown that co-assembly of eukaryote SAGs from several cells of the same population significantly increases the proportion of the genome that can be recovered^16,17^.

As with prokaryotes, the high diversity of single celled eukaryotes in marine ecosystems is problematic for metagenomic or metatranscriptomic approaches alone. Without assembled genomes it is difficult to assign functional genes to species^38^. In addition, most marine eukaryotes have not been cultivated, especially the heterotrophic types^39^, so traditional genome sequencing is not a viable option. The Marine Microbial Eukaryote Transcriptome Sequencing Project sought to sequence the transcriptomes of about 650 important marine microbial eukaryotes, and has produced a powerful sequence dataset^30^. It is limited, however, in only including cultured types with an emphasis on phototrophs. Keeling *et al*.^30^ acknowledge that single cell genomics will play an important complementary approach to gain understanding of these diverse protists. The approach is a powerful complement to environmental metatranscriptomics^38^.

For prokaryotes the high potential metabolic diversity of communities is well established, and advances in understanding the relationships between genetic diversity and ecosystem function is currently an area of active ecological research not only in environmental systems, but microbiomes of metazoans including humans. The high diversity of eukaryotic protists in the ocean is less well appreciated, but similarly enigmatic. Conventional knowledge has limited their metabolic or ecological function to phototrophy and heterotrophy. New evidence, however, reveals complexities of mixotrophy, endosymbiosis, and parasitism that could dominate the functions of marine protists. More intricate relationships based on small scale physical structuring, resource sharing, and chemical communication could be the basis of niche separation allowing the high diversity observed. Sequencing single eukaryotic cells sampled directly from the ocean as described here offers a way forward in deciphering who is doing what and how in the ocean.

## Supplementary information

Supplementary Material

Supplementary Table 2
