# Supplementary material for: Single cell genomics yields a wide diversity of small planktonic protists across major ocean ecosystems
Source: Sci Rep. 2019 Apr 15;9:6025. doi: 10.1038/s41598-019-42487-1 (PMC6465268; doi:10.1038/s41598-019-42487-1)
Supplement: Supplementary file 1 — Supplementary Material [file 41598_2019_42487_MOESM1_ESM.docx]

**Single cell genomics yields a wide diversity of small planktonic protists across major ocean ecosystems**

M. E. Sieracki, N. J. Poulton, O. Jaillon , P. Wincker, C. de Vargas, L. Rubinat-Ripoll, R. Stepanauskas, R. Logares, R. Massana

**Supplementary Material**

Supplementary Table S1. Summary of MDA plate performance from all 8 samples in this study. There are 315 one-cell wells per plate (other wells are blanks and controls). Good MDA wells are defined by Cp values less than 14h in the rtMDA data. SAGs identified are those with good quality 18S sequence. Yield is the percent of one-cell wells containing identified SAGs (good quality 18S sequence).

|  | Aplastidic | Plastidic | Total |
| --- | --- | --- | --- |
| Number of plates | 14 | 15 | 29 |
| One-cell wells | 4410 | 4725 | 9135 |
| Good MDA wells | 2288 | 2109 | 4397 |
| SAGs identified | 647 | 342 | 989 |
| Yield per plate | 14.7% | 7.2% | 10.8% |

Supplementary Table S2. List of all SAGs with good quality 18S sequence.

(see Excel file)

Supplementary Table 3. Comparison of richness, evenness and diversity of the protist communities across the stations sampled. These are calculated at the level of our defined groups. Plastidic and aplastidic forms were combined.

| Sample | Richness (S) | Evenness (J) | Diversity (H) |
| --- | --- | --- | --- |
|  |  |  |  |
| Stn23 | 14 | 0.851 | 2.246 |
| Stn39 | 14 | 0.782 | 2.065 |
| Stn41D | 17 | 0.822 | 2.329 |
| Stn41S | 18 | 0.831 | 2.402 |
| Stn46 | 14 | 0.813 | 2.146 |
| Stn47 | 16 | 0.876 | 2.428 |
| Stn48 | 16 | 0.886 | 2.456 |
| Stn51 | 15 | 0.815 | 2.797 |

The SAG composition of the different stations was sub-sampled to the lowest number of SAGs per station (52 counts) using rrarefy in Vegan (1). In the subsampled SAG table, fungi was not present. Using the subsampled table, we calculated Richness (number of taxonomic ranks/classes per station) and Evenness (Pielou’s J  =  H/log(S)). All analyses were run in the R statistical environment (2).

1. Oksanen, J., et al., *vegan: Community Ecology Package. R package version 1.15-0.* 2008.

2. R-Development-Core-Team, *R: A language and environment for statistical computing.* 2008, Vienna, Austria: R Foundation for Statistical Computing.

**Supplementary Figure Legends**

Figure S1. Maps of station locations in a. the Adriatic Sea, and b. the Indian Ocean. Map data: Google Earth, USGS, Orion-ME, US Dept. of State Geographer, SIO, NOAA, US Navy, NGA, GEBCO.

Figure S2. Comparison of flow cytometric detection of plastidic (a, b) and aplastidic (c, d) protists live (a, c) and cryopreserved with glycine betaine (b, d), for a coastal Maine sample. The red dots in the plastidic cytograms (a, b) are *Synechococcus*, determined by phycoerythrin fluorescence, and the blue dots are the plastidic protists. The polygons in the aplastidic cytograms (c, d) show the count region used for comparing live and cryopreserved samples.

Figure S3. Comparison of PCR primer performance for one plate each of a) plastidic SAGs, and b) aplastidic SAGs from Station 41. Venn diagrams show the numbers of SAGs with successful PCR product for each of the three primers: V4, V9, and Euk528/B.


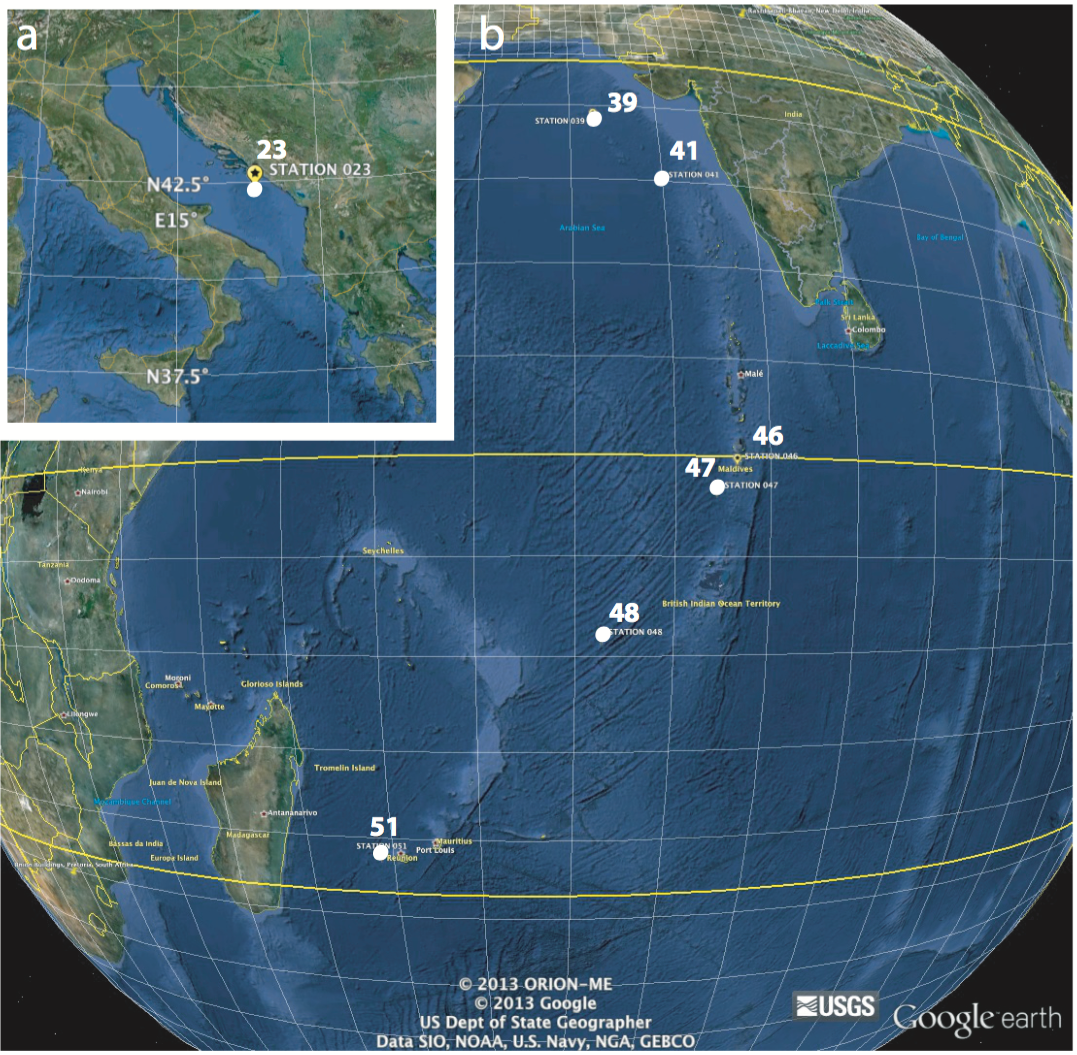


Figure S1. Sieracki et al.

Figure S2. Sieracki et al.


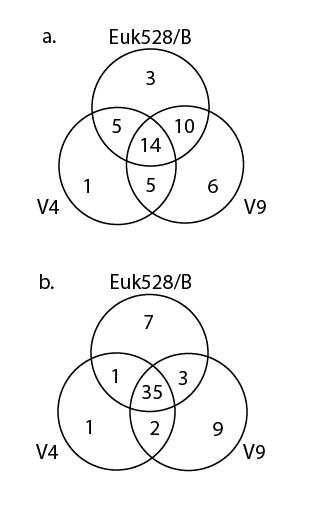


Figure S3. Sieracki et al.
